# Assessing of executive functions in daily life in preterm children aged 3–4 years old from the “Behavior Rating Inventory of Executive Function—Preschool version” questionnaire

**DOI:** 10.3389/fped.2023.999100

**Published:** 2023-07-26

**Authors:** Magali Reynold de Seresin, Arnaud Roy, Camille Theveniaut, Justine Le Goff, Coline Chopin, Valérie Rouger, Jean-Christophe Roze, Cyril Flamant, Jean-Baptiste Muller

**Affiliations:** ^1^ Pediatric and Neonatal Intensive Care Unit, Nantes University Hospital, Nantes, France; ^2^ Réseau “Grandir Ensemble”, Nantes University Hospital, Nantes, France; ^3^ Department of Psychology, Psychology Laboratory, University of Angers, Angers, France; ^4^ Reference Center for Learning Disabilities, Nantes University Hospital, Nantes, France; ^5^ Univ Angers, Nantes Université, LPPL, SFR CONFLUENCES, Angers, France; ^6^ Epidemiologie Clinique, Centre d'Investigation Clinique (CIC004), Nantes University Hospital, Nantes, France

**Keywords:** executive functions, preterm, preschool, parent, questionnaire

## Abstract

**Background:**

Executive functions (EFs) are a set of neuropsychological skills permitting solving problems in a new situation by regulating action, behavior, and emotional response. As cerebral maturation remains vulnerable in preterm children, a higher risk of developing cognitive disorders including EFs exist compared to term children.

**Aims:**

The aim of this study was to estimate the incidence of preschool EF impairments through proxy reports in children born preterm before 34 weeks of gestational age using the Behavior Rating Inventory of Executive Function—Preschool (BRIEF-P) version. Secondary aims were to report neonatal, child, or socioeconomic factors associated with EF disorders.

**Results:**

Parents of 357 children born preterm aged 3–4 years old completed the BRIEF-P version. Impairment in EFs was clinically significant for 13.5% of preterm children (*n* = 47; 95% CI = 0.10–0.18) compared to 5.1% in term children. A low parental socioeconomic level was significantly associated with impaired parent-rated EF (19.1% vs. 5.3%, *p *= 0.003).

**Conclusions:**

Proxy reports of EF impairment are about twice as frequent as in term children. EF difficulties are not related to neonatal or child severity factors in contrast with the parental socioeconomic level. Using a parent-rated questionnaire may be a useful and easy tool to identify early the daily life impact of EF disorders on clinical follow-up of preterm children.

This study was recorded in the Clinical Trials Register under identifier NCT03700463.

## 1. Introduction

Improvement in perinatal caregiving increases the survival rates of very preterm born infants (1). However, cerebral maturation remains vulnerable in these preterm infants, with a high risk of developing neurodevelopmental disorders including motor skill disorders, such as cerebral palsy (2, 3), cognition disorders (4, 5), and learning disability (6), and behavior disorders, such as attention-deficit/hyperactivity disorder (5, 7).

Executive functions (EFs) are a set of skills permitting solving problems in a new situation by regulating action, behavior, and emotional response. These abilities include inhibition (inhibiting behavior and impulse control), shifting (moving between activities or aspects of a problem), working memory (focusing on and holding information in one's mind to achieve a goal), planning (coordinating and planning current and future task requirements), and emotional control (regulating emotions). These functions are distinguished from other cognitive abilities such as language or memory since they are high-level and top-down control functions (8). In this way, EFs regulate foundational cognitive abilities engaged in executive task completion such as language or visual/spatial perception. At preschool age, EFs and foundational abilities are still developing with interindividual variation. Distinguishing EFs from foundational cognitive abilities remains challenging. However, implicit, bottom-up mechanisms such as associative and reinforcement learning could also play a core role in shaping cognitive control (9). EFs pertain to prefrontal brain networks but also to a larger distributed system of regions including other cortical and subcortical structures, such as the thalamus and the inferior parietal cortices (10–13), with maturation from early childhood to adulthood (14). Several studies demonstrate that early focal brain injury could impair EF development (15, 16). Neuropsychological studies have reported impairments of cognition disorders including EFs in very preterm infants compared to term-born children, especially for attention control and working memory for preschool-age children (17, 18).

EF assessment is usually based on performance-based tools and/or parent-reported everyday life. EF performance-based assessment tools such as the Developmental Neuropsychological Assessment battery (NEPSY-II) require time and trained professionals, which is not easy to perform in routine medical clinical practice, and are weakly correlated with parents’ real-life reports, especially in the preterm population (19–21). Indeed, EF performance-based assessment tools may underestimate real-life EF difficulties, especially in preterm children (21). Barkley proposed that performance-based EF assessments in clinical settings mostly evaluate the basic and momentary child EF abilities, whereas EF questionnaires are better at assessing the more complex adaptive EFs in real daily life (22). On the basis of these criticisms, Gioia et al. implemented a tool to assess executive functioning in everyday life: the “Behavior Rating Inventory of Executive Function” (23), in the form of a questionnaire completed by parents or the teacher of the child, allowing simple use in everyday practice (24, 25). The “Behavior Rating Inventory of Executive Function—Preschool (BRIEF-P) version” (26) allows EF assessment for children from 2 to 5 years and 11 months via 63 items related to behavior and daily living activities. The French version was performed by Roy and Le Gall (27).

The aim of this study was to estimate the impact of the incidence of EF disorder on daily life in preterm infants born less than 34 weeks of gestational age (GA), aged 3 to 4 years old, using the BRIEF-P version completed by the parents. Secondary aim of the study were to report factors associated with EF disorders among neonatal, child or socioeconomoc factors.

## 2.1. Materials and methods

### 2.1. Participants

The eligible participants were surviving preterm children born before GA of 34 weeks between July 2014 and the end of December 2015, aged 3 to 4 years old, and enrolled in the regional “Loire Infant Follow-up Team” (LIFT) cohort (28).

Patients suffering from congenital injury, including nervous system malformations, congenital heart diseases, digestive system abnormalities, congenital lung diseases, or genetic diseases, or whose parents do not have a complete understanding of written and oral French were excluded.

This study was approved by the Ethics Committee of the Persons Protection Committee (registered 1852, ID-RCB 2018-A01528-47) in July 2018. This work with respect to the reference methodology MR003 was registered at the French data protection authority named CNIL (“Commission Nationale de l’Informatique et des Libertés”).

This study was recorded in the Clinical Trials Register under identifier NCT03700463.

### 2.2. Questionnaire reports

All eligible patients included in the LIFT cohort, aged from 3 to 4 years old during study period, were contacted by informative letters. Parental consent was obtained. When the BRIEF-P questionnaire was not returned by parents after 1 month, a reminder was given by phone or email. Completed BRIEF-P parental questionnaires were recorded on online Hogrefe software.

To assess the behavioral manifestation of EF in everyday life, parents completed the BRIEF-P questionnaire. Items from the BRIEF-P questionnaire have been adapted from the “Behavior Rating Inventory of Executive Function” by Gioia et al. (23), based on clinical observations. The BRIEF-P questionnaire comprises five subscales: inhibition, shifting, emotional control, working memory, and planning/organization. These subscales can be summed to create the Global Executive Composite (GEC). GEC and subscales are age- and sex-normed to *T*-scores. The median *T*-score distribution is 50, and a score above 65 (+1.5 SD) is recognized to classify child participants as being in the clinical range (*T*-score at or above 65) vs. the nonclinical range (*T*-score less than 65). The answers to the questions are “Never,” “Sometimes,” or “Often” and are coded as ordinal variables (1 as “never”; 2 as “sometimes”; 3 as “often”). About 15 min are necessary to complete the BRIEF-P questionnaire. Incoherence and negativity scores are screened to express the validity of parental responses. The incoherence score increases if responses to similar items are not rated equally. An incoherence score at or above 9 for the parental BRIEF-P questionnaire is considered invalid and uninterpretable and is therefore excluded from statistical analysis. Negativity score measures the extent to which the person answering questionnaire items rates responses in an unusually negative way. The negativity score increases if there is a negative perception of the child, so it can be assumed that responses provided by respondents are considered pathological within the same domain in an excessive way. A negativity score at or above 4 should challenge the clinician, who will have to check whether other test responses confirm a major dysexecutive medical condition or whether it is an unusually negative assessment of the respondent.

### 2.3. Background variables

The LIFT cohort, used for neonatal and child data collection, is registered with the French data protection authority in clinical research (CNIL) under identifier 915452.

Neonatal variables collected were sex, GA at birth, *Z*-score of birth weight, birth head circumference, and *Z*-score of birth head circumference. We collected pathological events of the neonatal period such as mechanical ventilation, prolonged parenteral nutrition (as persistent perfusion above 35 weeks of GA), abnormal electroencephalogram, bronchopulmonary dysplasia (BPD) defined as oxygen therapy for at least 28 days and/or respiratory support required at 36 weeks of GA, and major brain injury (as grade 3 and 4 intraventricular hemorrhage or periventricular leukomalacia). Data at discharge were also collected as weight, Z-score of weight, delta of the *Z*-score of weight, *Z*-score of head circumference, delta of the *Z*-score of head circumference, and breastfeeding status. Socioeconomic data included maternal and paternal educational level according to INSEE (“Institut National de la Statistique et des Etudes Economiques”) and/or medicosocial care status. We defined three parental educational levels as high, intermediate, and low. High parental educational level was defined as at least one parent holding a diploma greater than or equal to 3 years after a bachelor’s degree. Low parental educational level was defined as both parents holding a diploma below a bachelor’s degree or “Couverture Medicale Universelle” (CMU) beneficiaries. Intermediate parental educational level was defined as neither high nor low level.

### 2.4. Statistical analysis

Data were collected using Excel (Microsoft Systems, Redmond, Washington, USA).

We compared socioeconomic, neonatal, and at-discharge data for included and eligible populations. Continuous quantitative variables were categorized using tercile methods, and qualitative variables were encoded in terms of absence/presence. Pearson's chi-square tests, with Yates correction when appropriate, were used to test the association of each variable with the primary outcome, and univariate logistic regression was performed. Correlation and covariance matrices were systematically analyzed to identify variable interlinking and avoid data circularity. Continuous variables were analyzed by ANOVA. We then performed a multivariable logistic regression, adjusting for social factors and factors associated with outcomes. A *p*-value under 0.05 was considered statistically significant. Statistical analysis was performed by IBM SPSS Statistics for Windows.

## 3. Results

### 3.1. Participants’ characteristics

A multicenter prospective observational study was conducted at Pays de la Loire, France, between October 15, 2018 and June 15, 2019.

**Figure 1:**
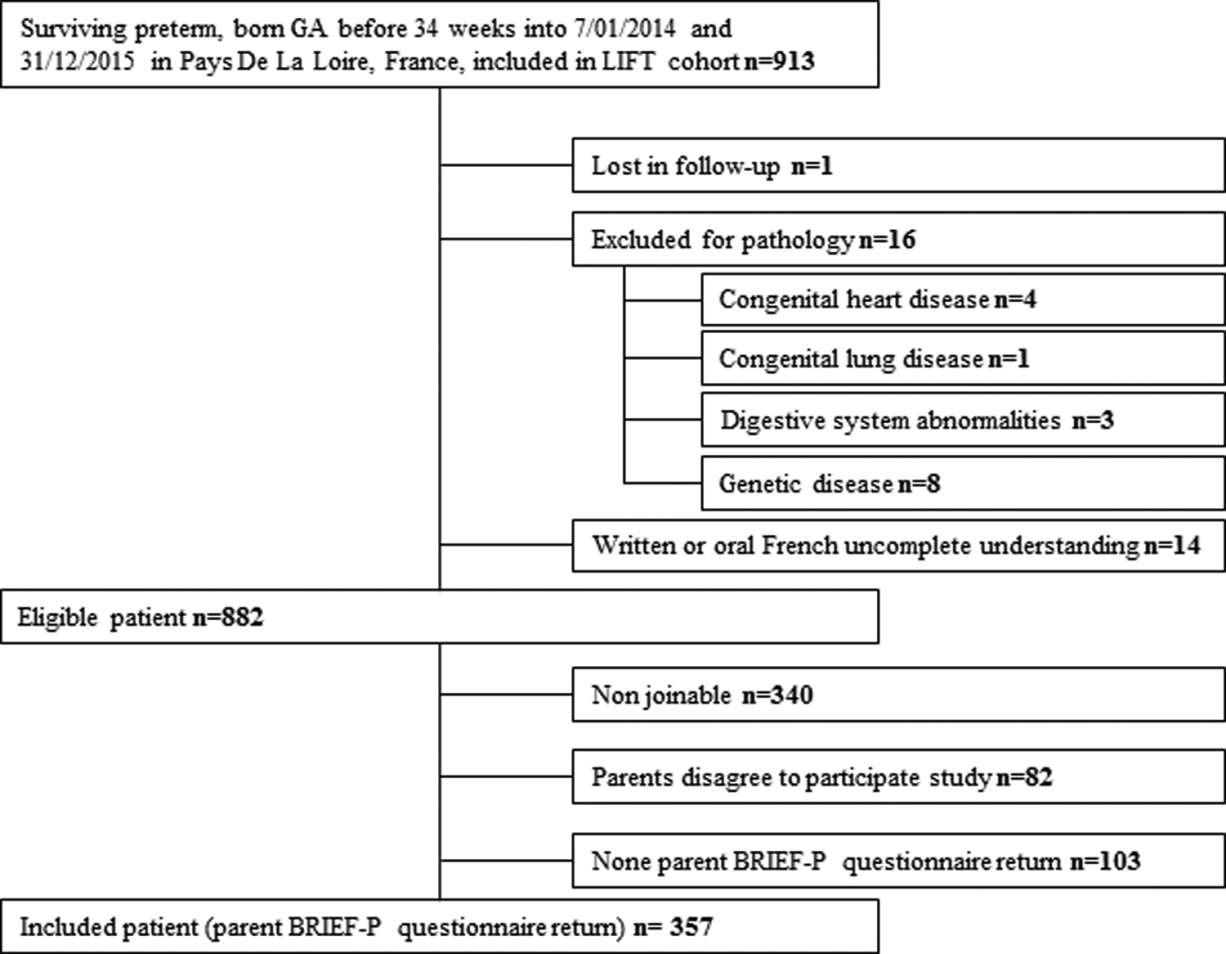
Flowchart.

Nine hundred and thirteen children were born between July 01, 2014 and December 31, 2015 and included in the LIFT cohort. Sixteen children were excluded for pathologies, and one was lost in follow-up. For 14 children, written or oral French understanding of parents was considered insufficient to complete the questionnaire. Eight hundred and eighty-two children were eligible, and their parents received the BRIEF-P questionnaire through mail. Only 357 patients returned the BRIEF-P questionnaire and were included in the study. The other patients were lost after failed phone, mail, and email contact attempts. A flowchart is presented in Figure 1.

A comparison of the demographics and neonatal medical conditions of the included population and the eligible population is given in Table 1. The included and eligible groups were similar for sex, gestational age, birth measurements, and weight measure at term. There was a significant difference in breastfeeding at discharge. Likewise, the *Z*-score of head circumference was significantly different between groups since the included population had better head circumference growth. In other words, the included population was selected among parents with high educational levels (28.3% vs. 14.3% in the eligible population, *p* < 0.001).

**Table 1:** Demographics and neonatal medical conditions of included population and study eligible population from the LIFT cohort of preterm born from 01 July 2014 to 31 December 2015.

	Total *n* = 882	Included population *n* = 357	Eligible population *n* = 525	*p*-Value
Neonatal data				
Boys, *n* (%)	478 (54.2)	192 (53.8)	286 (54.5)	0.839
Girls, *n* (%)	404 (45.8)	165 (46.2)	239 (45.5)	0.839
Gestational age (weeks) (mean, SD)	30.5 (2.32)	30.4 (2.23)	30.6 (2.38)	0.301
24–25, *n* (%)	31 (3.5)	12 (3.4)	19 (3.6)	
26–27, *n* (%)	100 (11.3)	37 (10.4)	63 (12)	
28–29, *n* (%)	125 (14.2)	59 (16.5)	66 (12.6)	
30–31, *n* (%)	228 (25.8)	112 (31.4)	116 (22.1)	
32–33, *n* (%)	398 (45.1)	137 (38.4)	261 (49.7)	
Z-score of birth weight, mean (SD)	−0.21 (1.05)	−0,25 (1.02)	−0,18 (1.07)	0.348
Birth head circumference (cm), mean (SD)	33.3 (1.81)	33.4 (1.87)	33.2 (1.77)	0.238
*Z*-score of birth head circumference, mean (SD)	−0.27 (0.94)	−0.26 (0.93)	−0.27 (0.94)	0.944
Mechanical ventilation, *n* (%)	360 (40.8)	149 (41.7)	211 (40.2)	0.647
Perfusion time at GA >35 weeks, *n* (%)	29 (3.3)	10 (2.8)	19 (3.6)	0.504
Abnormal electroencephalogram, *n* (%)	84 (9.5)	27 (7.6)	57 (10.9)	0.102
Bronchopulmonary dysplasia, *n* (%)	84 (9.5)	33 (9.2)	51 (9.7)	0.467
Major brain injury, *n* (%)	29 (3.3)	13 (3.6)	16 (3.0)	0.627
Clinical data at discharge				
Gestational age (weeks), mean (SD)	37.6 (2.11)	37.6 (1.98)	37.6 (2.20)	0.872
Weight (g), mean (SD)	2,643 (525)	2,625 (489.9)	2,655 (548.9)	0.432
*Z*-score of weight at term, mean (SD)	−0.84 (0.90)	−0.88 (0.90)	−0.81 (0.90)	0.282
Delta *Z*-score of weight at term, mean (SD)	−0.59 (0.76)	−0.60 (0.77)	−0.58 (0.75)	0.687
*Z*-score of head circumference at term, mean (SD)	−0.22 (0.91)	−0.14 (0.96)	−0.28 (0.87)	0.048*
Delta *Z*-score of head circumference at term, mean (SD)	0.07 (0.91)	0.12 (0.94)	0.03 (0.88)	0.227
Breastfeeding, *n* (%)	186 (21.1)	94 (26.3)	92 (17.5)	0.002*
Parental educational level				
High, *n* (%)	176 (20)	101 (28.3)	75 (14.3)	<0.001*
Intermediate, *n* (%)	552 (62.6)	231 (64.7)	321 (61.1)	<0.001*
Low, *n* (%)	154 (17.5)	25 (7)	129 (24.6)	<0.001*

* *p* < 0.05.

### 3.2. Outcomes

The incoherence score was invalid for nine parental BRIEF-P questionnaires, leading to removing them from statistical analysis. Thereby, 348 parental BRIEF-P questionnaires were analyzed.

Impairment in EFs was clinically significant for 13.5% of preterm children (*n* = 47; 95% CI = 0.10–0.18), with the GEC T-score at or above 65.

**Figure 2:**
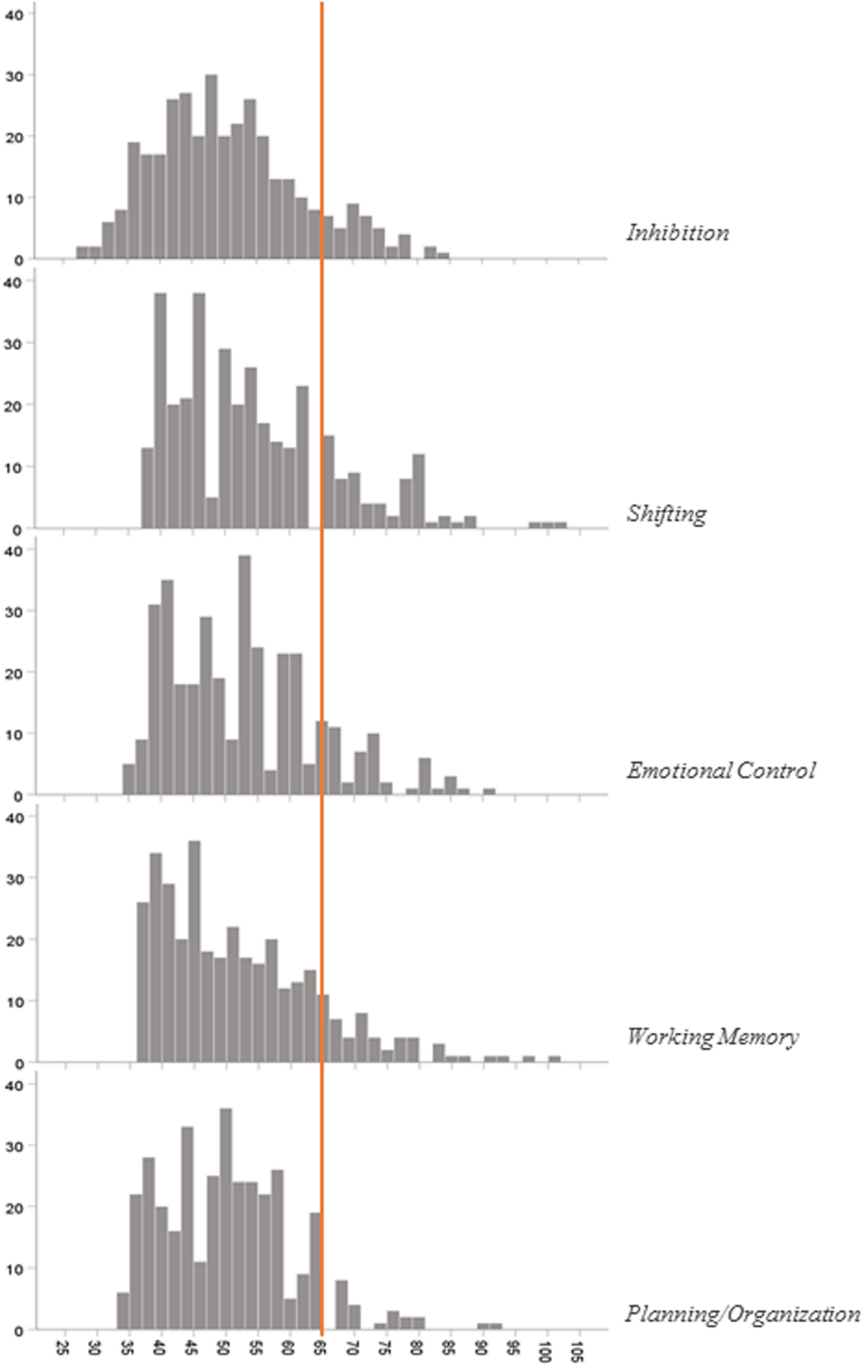
*T*-score distribution of BRIEF-P questionnaire subscales. A *T*-score at or above 65 is clinically significant.

The *T*-score distribution of five EF subscales is presented in Figure 2. The included preterm subscale *T*-score was in the clinical range of 12.1%, 20.4%, 14.1%, 12.4%, and 6.3%, respectively, for inhibition, shifting, emotional control, working memory, and planning/organization.

### 3.3. Secondary aims

Univariate analysis results are presented in Table 2, revealing that neonatal medical conditions were not associated with impaired parent-rated EFs. By contrast, the socioeconomic condition was associated with clinically significant impairment of EF. A low parental educational level was significantly associated with impaired parent-rated EF (19.1% vs. 5.3%, *p* = 0.003).

**Table 2:** Effects of demographics and neonatal medical conditions on parent-rated executive functions (*n* = 348).

	Clinical range of GEC *n* = 47	Nonclinical range of GEC *n* = 301	*p*-value
Neonatal data			
Boys, *n* (%)	22 (46.8)	165 (54.8)	0.306
Girls, *n* (%)	25 (53.2)	136 (45.2)	0.306
Gestational age (weeks), mean (SD)	30.1 (2.34)	30.5 (2.20)	0.249
Z-score of birth weight, mean (SD)	−0.41 (0.95)	−0.21 (1.02)	0.222
Z-score of birth head circumference, mean (SD)	−0.30 (0.79)	−0.26 (0.94)	0.797
Mechanical ventilation, *n* (%)	22 (46.8)	122 (40.5)	0.416
Perfusion time at GA >35 weeks, *n* (%)	3 (6.4)	6 (2)	0.078
Abnormal electroencephalogram, *n* (%)	6 (12.8)	21 (7)	0.168
Bronchopulmonary dysplasia, *n* (%)	5 (10.6)	26 (8.6)	0.887
Major brain injury, *n* (%)	3 (6.4)	10 (3.3)	0.303
Clinical data at discharge			
Gestational age at discharge (weeks), mean (SD)	37.8 (1.96)	37.5 (2.00)	0.485
*Z*-score of weight at term, mean (SD)	−1.10 (0.77)	−0.84 (0.91)	0.079
Delta *Z*-score of weight at term, mean (SD)	−0.68 (0.63)	−0.60 (0.80)	0.549
*Z*-score of head circumference at term, mean (SD)	−0.40 (0.79)	−0.09 (0.99)	0.077
Delta *Z*-score of head circumference at term, mean (SD)	−0.06 (0.78)	0.15 (0.95)	0.227
Breastfeeding, *n* (%)	10 (21.3)	81 (26.9)	0.414
Parental educational level			
High, *n* (%)	10 (21.3)	87 (28.9)	<0.003*
Intermediate, *n* (%)	28 (59.6)	198 (65.8)	<0.003*
Low, *n* (%)	9 (19.1)	16 (5.3)	<0.003*

* *p* < 0.05.

Multivariate analysis confirms that socioeconomic status is significant for impaired parent-rated EF, with the low parental educational level being 4.08 times more likely to have impaired behavior problems (95% CI = 1.22–13.53; *p* = 0.02). Neither any neonatal nor discharge medical factor was associated with EF disorders in multivariate analysis. Odds ratios predicting impaired parent-rated EF are presented in Table 3.

**Table 3:** Multivariate logistic regression predicting impairment in parent-rated EF (BRIEF-P GEC score).

	*Β*	SE	OR	95% CI	*p*-value
Sex (F)	0.23	0.36	0.36	0.62–2.57	0.52
Gestational age (in weeks)					
24–25	1.18	0.72	3.26	0.80–13.37	0.10
26–27	−0.06	0.70	0.94	0.24–3.69	0.93
28–29	0.30	0.50	1.34	0.50–3.61	0.56
30–31	0.42	0.43	1.52	0.65–3.55	0.33
32–33	0		1		
*Z*-score of birth weight	−0.16	0.16	0.86	0.62–1.18	0.34
Nonperfusion time at GA >35 weeks	−0.97	0.81	0.38	0.08–1.84	0.23
Parental educational level					
High	0		1		
Intermediate	0.21	0.42	1.24	0.54–2.84	0.61
Low	1.41	0.61	4.08	1.23–13.53	0.02*

* Significance at *p* < 0.05.

## 4. Discussion

The aim of the current study was to assess the impact of the incidence of EF disorders on daily life through a proxy report in preterm children aged 3–4 years old. Our results apply to the included population with high socioeconomic status and fewer medical risk factors than the eligible population. Nevertheless, more than 13% of parents report global EF difficulties in the everyday life of their children. Neonatal factors such as gestational age or birth weight were not related to EF disorders, but a low parental socioeconomic level was significantly associated with impaired parent-rated EF disorders.

Compared to term children, impaired parent-rated EF in our preterm preschool cohort is about twice as common (estimated in the French and US control populations at approximately 5.1% and 6.5%, respectively) (27). Scientific literature is still scarce in assessing everyday life EF disorders at preschool age in preterm children. Meether, in a small sample of 48 preterm born ≤32 weeks gestation, reported no more than 6% of children with a GEC score in the clinical range at 4 years (29). However, our results emphasize the importance of assessing everyday life EF disorders in preterm children as early as 3–4 years old. Even if at preschool age, parents’ complaint is still moderate compared to parents’ complaints at school age (26), and this is related to the degree of increasing cognitive demands.

Subscale analysis in the current study pointed out that the most clinically relevant scores have been observed for the shifting subscale. Meether, in his preterm cohort aged 4–5 years old, reported the most clinically relevant score for the working memory subscale. However, their study population included children with a higher rate of brain injury than our study population, with a lower average gestational age at birth but a higher socioeconomic level (29). Indeed, not only medical but also social and environmental characteristics influence FE development, but this may be different (30). For instance, the working memory subscale as the central function may be associated with more severe cognitive impairment, while the shifting subscale may reveal more specifically isolated EF disorders in early childhood. Still, our result remains consistent with those in the literature because the executive function ability seems modulated by environmental and experience-related circumstances in early childhood (17, 29).

Socioeconomic level constituted risk factors for impaired parent-rated EF in our preschool preterm children. Different neurocognitive systems are not uniformly affected by the socioeconomic level. Previous studies on general population demonstrate that children from higher-social-risk backgrounds develop less skillful EFs (31). Recent EF models propose that implicit and reinforcement learning plays an important role in EF development (9). This stimulation may be missing with lower parental educational levels. Indeed, preschool EF parental complaint is more frequent in this social group. In preterm children, low socioeconomic level as a predictor of long-term cognitive disabilities needs no further proof (32, 33). However, very few studies have explored the socioeconomic level and EF skills in the preterm cohort, especially at preschool age. O'Meagher identified that higher social risk is independently associated with parent-rated EF skills. More precisely, the strongest predictor of EF skills in this preterm preschool cohort is the main caregiver's educational level. These important concordant results have to be underlined. More than medical factors, such as gestational age or birth weight, the surrounding social context could be the main factor associated with parents' everyday life EF complaint (34).

Ideally, performance-based standardized EF tests should be performed and compared to daily life questionnaires. It is known that they are not always correlated, especially at preschool age (16). Therefore, as a gradual and continued process of maturation, identifying early impaired behaviors may be challenging at this stage; as time goes by, increased cognitive request may reveal EF impairments (29). A longitudinal assessment starting from 3–4 years to adulthood would help understand EF development trajectory in preterm children and put things into perspective from EF developmental delay to EF impairment.

Some limitations of the current study are related to the characteristics of the included population. Indeed, responders constituted a low-risk population of long-term impairment with a higher socioeconomic level, larger head circumference at discharge, and more often breastfeeding (33, 35, 36). Preschool parent-related EF impairment in preterm children is probably underestimated to a great extent. Yet complaints concern at least 13% of parents of preterm as soon as 3–4 years old. The strength of this study is large number of patients. To our knowledge, this study is the largest preterm cohort of parent-related preschool EFs.

## 5. Conclusion

At early preschool age, preterm children displayed EF disorders twice as frequently as the term population. The social environment played a crucial role in parent-reported impaired EF complaint. A longitudinal exploratory risk factor analysis follow-up of EF assessment would help understand EF development of preterm children.

## Data Availability

The raw data supporting the conclusions of this article will be made available by the authors without undue reservation.
